# The mTOR kinase inhibitors polarize glioma-activated microglia to express a M1 phenotype

**DOI:** 10.1186/1742-2094-11-125

**Published:** 2014-07-23

**Authors:** Lucia Lisi, Emilia Laudati, Pierluigi Navarra, Cinzia Dello Russo

**Affiliations:** 1 Institute of Pharmacology, Catholic University Medical School, L.go F Vito 1, 00168 Rome, Italy

## Abstract

**Background:**

Increased activation of mammalian target of rapamycin (mTOR) is observed in numerous human cancers. Recent studies on the glioma kinome have identified several deregulated pathways that converge and activate mTOR. The available evidence on the role of microglia in CNS cancers would suggest a dual role, a tumoricidal role and -on the contrary- a role favoring tumor growth.

**Methods:**

In the present paper, we have compared the effects of μM concentrations of rapamycin (RAPA) and its analog, RAD001 (RAD), on activated microglia; the latter was obtained by exposing cells to conditioned medium harvested either from inflammatory activated glioma cells (LI-CM) or from glioma cells kept under basal conditions (C-CM).

**Results:**

Here we show that the inhibition of mTOR polarizes glioma-activated microglial cells towards the M1 phenotype, with cytotoxic activities, preventing the induction of the M2 status that promotes tumor growth. In fact RAPA and RAD significantly increased iNOS expression and activity, while on the same time significantly reducing IL-10 gene expression induced by C-CM, thus suggesting that the drugs prevent the acquisition of a M2 phenotype in response to glioma factors promoting a classic M1 activation. Similar results were obtained using the conditioned media obtained after glioma stimulation with LPS-IFNγ (LI-CM), which was found to induce a mixture of M1 and M2a/b polarization phenotypes. In these conditions, the inhibition of mTOR led to a significant up-regulation of iNOS, and in parallel to the down-regulation of both ARG and IL-10 gene expression.

**Conclusions:**

These data suggest that mTOR inhibition may prevent glioma induced M2 polarization of microglial cells and increase their cytotoxic potential, possibly resulting in antitumor actions.

## Introduction

The mammalian target of rapamycin (mTOR) is a conserved serine/threonine protein kinase involved in the regulation of multiple intracellular processes such as mRNA transcription and translation, ribosomal biogenesis, lipid biosynthesis, energy metabolism, autophagy and cytoskeletal organization [1]. Within the cells two distinct complexes, namely mTORC1 and mTORC2, are expressed, that are associated to different partners [2]. Increased activation of mTORC1 is observed in numerous human cancers due to gain-of-function mutations in oncogenes (PI3K, AKT or Ras) and/or loss-of-function mutations in tumor suppressors (PTEN, LKB1, or TSC1/2), upstream regulators of mTORC1 [3]. These mutations provide cancer cells with a selective growth advantage in comparison to normal cells [4]. On the other hand, mTORC2 is associated with cell adhesion and migration, thus mainly with tumor invasion of normal tissue [5].

Glioblastomas (GBM, World Health Organization (WHO) IV astrocytomas) are the most common and aggressive primary central nervous system (CNS) tumors, originating from glial cells and characterized by morphological and genetic complexity [6]. Current therapeutic strategies (that is, aggressive surgical resection combined with radiation and chemotherapy) are largely ineffective; tumors often recur and are invariably fatal, with a median survival of 12 to 15 months from diagnosis [7]. Recent studies on the glioma kinome have identified several deregulated pathways that converge and activate mTOR [8].

Despite the central role of mTOR in glioma biology, mTORC1 inhibitors, like rapamycin (RAPA) and its analogs (rapalogs), have been shown to have limited efficacy in clinical trials [8], with the exception of RAD001 in pediatric patients affected by subependymal giant-cell astrocytomas (WHO I astrocytomas) [9]. The reasons for the limited clinical success of rapalogs (in glioma but also in major solid tumors) have not been clarified but are likely related to the inhibition of a large number of mTORC1-regulated signaling systems normally involved in tumor suppression, such as the activation of receptor tyrosine kinases (RTKs), PI3K-Akt signaling, and the Ras-ERK pathway [10]. Moreover, rapamycin is unable to inhibit all the downstream targets of mTORC1 and to significantly suppress mTORC2 signaling [11]. In order to by-pass these limitations, alternative strategies have been explored in the past few years and a number of ATP-competitive mTOR inhibitors have been developed, which block both mTORC1 and mTORC2 activity. Interestingly, because of the high sequence homology between mTOR and PI3K, some compounds originally identified as PI3K inhibitors were later shown to inhibit mTOR as well [10]. Unlike RAPA, which is a specific allosteric inhibitor of mTORC1, these ATP-competitive inhibitors target the catalytic site of the enzyme, thus promoting a broader, more potent and sustained inhibition of mTOR, and preventing the activation of PI3K/Akt caused by the de-repression of negative feedbacks [12]. At present, ClinicalTrials.gov lists 299 ongoing studies on mTOR and cancer, 17 being studies on mTOR and glioma, mostly testing the dual inhibition of mTORC1 and mTORC2.

Recently, a direct role of mTOR in the modulation of glial functions has also been described. Data from our group and others support the notion that mTOR is involved in glial proinflammatory activation [13-15]. In particular, we have demonstrated that mTOR inhibitors reduce iNOS expression and activity induced by cytokines, but not those induced by lipopolysaccharide (LPS), in primary rat microglia [13]. Among glioma-infiltrating cells, microglia/macrophages represent the largest population, contributing to the total tumor mass by at least one third [16]. Under pathological conditions, these cells do not constitute a uniform cell population, but rather comprise a family of cells with diverse phenotypes and opposite biological activities: beneficial or, on the contrary, detrimental [17]. Macrophages and activated microglia, the latter defined as CNS macrophages, can be broadly divided into classically activated M1 cells, with cytotoxic properties, and alternatively activated M2 cells, with phagocytic activities. Macrophages infiltrating tumor tissues are polarized by tumor-derived and T-cell-derived cytokines towards an M2 phenotype [18]. In general, it is now well-established that tumor-associated macrophages promote tumor growth and progression [19]. As far as microglia and gliomas are specifically concerned, under the influence of glioma M2 microglia release several classes of molecules that foster glioma growth [20], progression and inflammatory activation [21] as opposed classically activated microglia, the M1 phenotype, release inflammatory products, such as nitrites, IL-10 and urea, that may exert detrimental effects on glioma cells. We have recently found that microglial cells undergo a different pattern of activation depending on the stimulus; in the presence of inflammatory activated glioma-derived factors, that is, a condition mimicking the late stage of pathology, microglial cells appear as a mixture of polarization phenotypes (M1 and M2a/b). At variance, microglia exposed to basal glioma-derived factors, that is, a condition resembling the early stage of pathology, show a more specific pattern of activation, with increased M2b polarization status [22].

The tuberous sclerosis complex (TSC)-mTOR pathway regulates macrophage polarization [23]; in particular, it seems that mTOR activation leads to M2 polarization. In fact, bone morphogenetic protein-7 (BMP-7) mediates monocyte polarization into M2 macrophages through the activation of the PI3K-AKT-mTOR pathway [24] and RAPA, by blocking mTOR, and unbalances the polarization of human macrophages towards the M1 status [25].

In the present paper, we have compared the effects of μM concentrations of RAPA and its analog, RAD, on activated microglia, in the attempt to block both mTORC1 and mTORC2 functions [26]. Microglial activation was obtained by exposing cells to conditioned medium harvested either from inflammatory activated glioma cells (LI-CM) or from glioma cells kept under basal conditions (C-CM) [20]. Consistent with the findings in peripheral macrophages, here we show that the inhibition of mTOR polarizes glioma-activated microglial cells towards the M1 phenotype endowed with cytotoxic activities, thus preventing the induction of the M2 status that promotes tumor growth.

## Methods

### Materials

Cell culture reagents (DMEM, DMEM-F12 and FCS) were from Invitrogen Corporation (Paisley, Scotland). Antibiotics were from Biochrom AG (Berlin, Germany). The rat recombinant IFNγ was purchased from Endogen (Pierce Biotechnology, Rockford, IL, USA). Bacterial endotoxin LPS (*Salmonella typhimurium*) was from Sigma-Aldrich (St Louis, MO, USA). β-actin (clone AC-74) mouse monoclonal antibody was from Sigma Aldrich; rabbit polyclonal anti-phospho (ser-2448) mTOR were purchased from Novus Biological (Littleton, CO, USA).

### Cell cultures

#### Microglia

Primary enriched cultures of rat glial cells, microglia and astrocytes, were prepared as previously described [27]. In particular, microglial cells were obtained by mixed cultures of cortical glial cells (at day 14 *in vitro*) by gentle shaking. Cells were plated in 96-well plates at a density of 3 × 10^5^ cells/cm^2^ using 100 μL/well DMEM-F12, containing 10% FCS and antibiotics. Under these conditions, the cultures were 95 to 98% CD11b-positive [28]. The use of animals for this experimental work has been approved by the Ethics Committee of the Catholic University Medical School.

#### C6 glioma cells

C6 glioma cells were passed once a week and were prepared as previously described [14]. Conditioned media from activated C6 glioma cells were generated following a protocol aimed to remove the proinflammatory stimulus from the medium [22]. Briefly, control-conditioned medium (C-CM) was obtained incubating C6 glioma cells for 4 h in plain medium, and after three washes with PBS in fresh plain medium for an additional 24 h. After this second period of incubation, the CM was collected, centrifuged to remove cellular debris and stored as C-CM. Similarly, LPS/IFNγ-conditioned medium (LI-CM) was prepared incubating C6 cells for 4 h with LI, followed after three washes with PBS by 24-h incubation in fresh plain medium. LI-CM was collected, centrifuged and stored after this second period of incubation, thus LI-CM did not contain the proinflammatory stimuli used to activate glioma cells. Both CM were stored at −80°C until the experiments on microglial cells were performed.

### Nitrite assay

Inducible nitric oxide synthase (iNOS) activity was assessed indirectly by measuring nitrite accumulation in the incubation media. Briefly, an aliquot of the cell culture media (80 μL) was mixed with 40 μL Griess Reagent (Sigma-Aldrich) and the absorbance measured at 550 nm in a spectrophotometric microplate reader (PerkinElmer Inc, Waltham MA, USA). A standard curve was generated during each assay in the range of concentrations 0 to 100 μM using NaNO2 (Sigma-Aldrich) as the standard. In this range, standard detection produced a linear distribution and the minimum detectable concentration of NaNO2 was ≥3.12 μM. In the absence of stimuli basal levels of nitrites were below the detection limit of the assay at all the time points studied.

### mRNA analysis in real-time PCR

Total cytoplasmic RNA was extracted using the RNeasy Micro kit (Qiagen, Hilden, Germany), which included 15 minutes of DNAse treatment. RNA concentration was measured using the Quant-iTTM RiboGreen® RNA Assay Kit (Invitrogen Corporation, Carlsbad, CA, USA). A standard curve in the range of 0 to 100 ng was run in each assay using 16S and 23S ribosomal RNA (rRNA) from *Escherichia coli* as standard and provided by the kit. Aliquots (0.15 μg) of RNA were converted to cDNA using random hexamer primers. Quantitative changes in mRNA levels were estimated by real time PCR (Q-PCR) using the following cycling conditions: 35 cycles of denaturation at 95°C for 20 s; annealing and extension at 60°C for 20 s, using the Brilliant III Ultra-Fast SYBR® Green QPCR Master Mix (Stratagene, La Jolla, CA, USA), except for Raptor gene expression (35 cycles of denaturation at 95°C for 20 s; annealing 64°C and extension at 60°C for 20 s, using the Brilliant II SYBR® Green QPCR Master Mix). PCR reactions were carried out in a 20-μL reaction volume in a MX3000P real-time PCR machine (Stratagene). Primers used for the evaluation of gene expression are reported in Table 1. Relative mRNA concentrations were calculated from the take-off point of reactions (threshold cycle, Ct) using the comparative quantitation method performed by Stratagene software and based upon the -ΔΔCt method [13].

**Table 1:** Primer sets used for Q-PCR analysis

**Genes**	**Forward primers**	**Reverse primers**	**Product length (Base pair)**
α-TUB	CCC TCG CCA TGG TAA ATA CAT	ACT GGA TGG TAC GCT TGG TCT	110
iNOS	CTG CAT GGA ACA GTA TAA GGC AAA C	CAG ACA GTT TCT GGT CGA TGT CAT GA	230
IL-10	CAG CTG CGA CGC TGT CAT CGA	GCA GTC CAG TAG ATG CCG GGT G	198
ARG1	TGC CCT CTG TCT TTT AGG GC	CCT CGA GGC TGT CCC TTA GA	165
Raptor	GCA GGC CTG GGA CCT TGC TG	CGA CAG GGC CAA GCT CAC CG	271
Rictor	AGC AGT GAT CCG AAA GGA GGG AAA	TAC TTG GAG TGC TGC CAG TGT CTT	199

### Cell viability measurement

Microglial viability was assessed by reduction of the tetrazolium compound (3-(4,5-dimethylthiazol-2-yl)-5-(4-sulfophenyl)-2H-tetrazolium, inner salt; MTS) contained in the CellTiter AQueous One Solution Reagent (Promega, Madison, WI, USA). For this assay cells were seeded in 96-well plates. At the end of the experimental procedure (46 h), 20 μL of MTS reagent were added to the cells that were further incubated for 2 h. Living cells bio-reduce yellow MTS into a purple soluble formazan product with an absorbance peak at 492 nm, that was read in a spectrophotometric microplate reader (PerkinElmer Inc, MA, USA).

### Cell proliferation assay

Microglial proliferation rate was determined using a nonradioactive proliferation assay (Exalpha Biological Inc, Maynard, MA, USA), used according to the manufacturer’s instructions. This ELISA measures the incorporation of 5-bromo-2-deoxyuridine (BrdU) into newly synthesized DNA of actively proliferating cells. Microglial cells were incubated in plain medium or C-CM, LI-CM for 48 h. BrdU solution was added 32 h later directly in the incubation medium, and cells were kept in the incubator for the remaining 16 h. At the end of the incubation time, medium was removed and cells were fixed. !BrdU immunoreactivity was measured using a specific primary antibody provided by the kit. Data are expressed as percentage of control values.

### Western immunoblot

The cells were lysed in RIPA buffer (1 mM EDTA, 150 mM NaCl, 1% igepal, 0.1% sodium dodecyl sulfate, SDS, 0.5% sodium deoxycholate, 50 mM Tris–HCl, pH 8.0) (Sigma-Aldrich) containing protease inhibitor cocktail diluted 1:250 (Sigma-Aldrich). The protein content in each sample was determined by Bradford’s method (Biorad, Hercules, CA, USA) using bovine serum albumin as the standard. A 10-μg aliquot of protein was mixed 1:2 with 2 × Laemmli Buffer (Biorad), boiled for 5 minutes, and separated through 7% polyacrylamide SDS gels. Apparent molecular weights were estimated by comparison to colored molecular-weight markers (Sigma-Aldrich). After electrophoresis, proteins were transferred to polyvinylidene difluoride membranes by semi-dry electrophoretic transfer (Biorad). The membranes were blocked with 10% (w/v) low-fat milk in TBST (10 mMTris, 150 mMNaCl, 0.1% Tween-20, pH 7.6) (Biorad) for 1 h at room temperature, and incubated in the presence of the primary antibody overnight with gentle shaking at 4°C. Primary antibodies for phosphorylated-mTOR (Novus biological), β-actin (Sigma-Aldrich) were used at the final concentration of 1:1,000. Primary antibodies were removed, membranes washed three times in TBST, and further incubated for 1 h at room temperature in the presence of specific secondary antibody, anti-rabbit for mTOR and anti-mouse for β-actin immunoglobulin G (IgG)-horseradish peroxidase (HRP)-conjugated (Sigma-Aldrich), diluted 1:15,000 and 1:10,000 respectively. Following three washes in TBST, bands were visualized by incubation in ECL reagents (Thermo Scientific, Rockford, Illinois, USA) and exposure to Hyperfilm ECL (GE Healthcare NY, USA). The same membranes were washed three times in TBST, blocked with 10% (w/v) low-fat milk in TBST for 1 h at room temperature and used for β-actin immunoblot.

### Data analysis

All experiments were performed using five to six replicates per each experimental group, and repeated at least three times. For the RNA analysis, samples were assayed in triplicates, and the experiments were repeated at least twice. Data were analyzed by one- or two-way analysis of variance (ANOVA) followed by Bonferroni post hoc test or by the unpaired *t*-test where appropriate. *P*-values <0.05 were considered significant.

## Results

**Figure 1:**
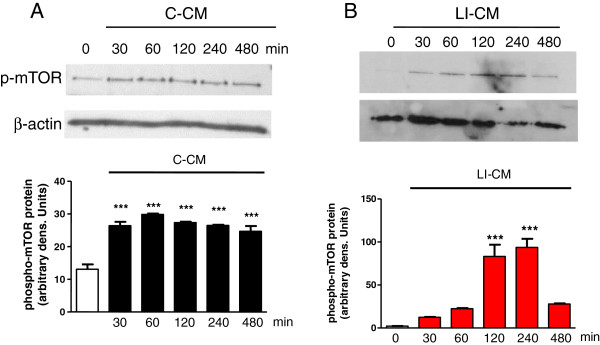
**Analysis of mammalian target of rapamycin (mTOR) phosphorylation during microglial activation.** Whole-cell lysates were prepared from microglial cells activated by control-conditioned medium (C-CM) **(A)** or CM from glioma cells activated with lipopolysaccharide (LPS)/IFNγ (LI-CM) **(B)**. Equal amounts of proteins were analyzed by western blot for phosphorylated mTOR kinase (p-mTOR), upper gel and were subsequently probed for β-actin, lower gel. Quantitation of densitometry where p-mTOR values are reflected relative to those for β-actin. Data are expressed as means ± standard error of the mean of n = 1 replicate for each group, each assayed in triplicate: representative of two different experiments. Data were analyzed by one-way analysis of variance followed by Bonferroni post hoc test. ***P <0.001 versus control.

**Figure 2:**
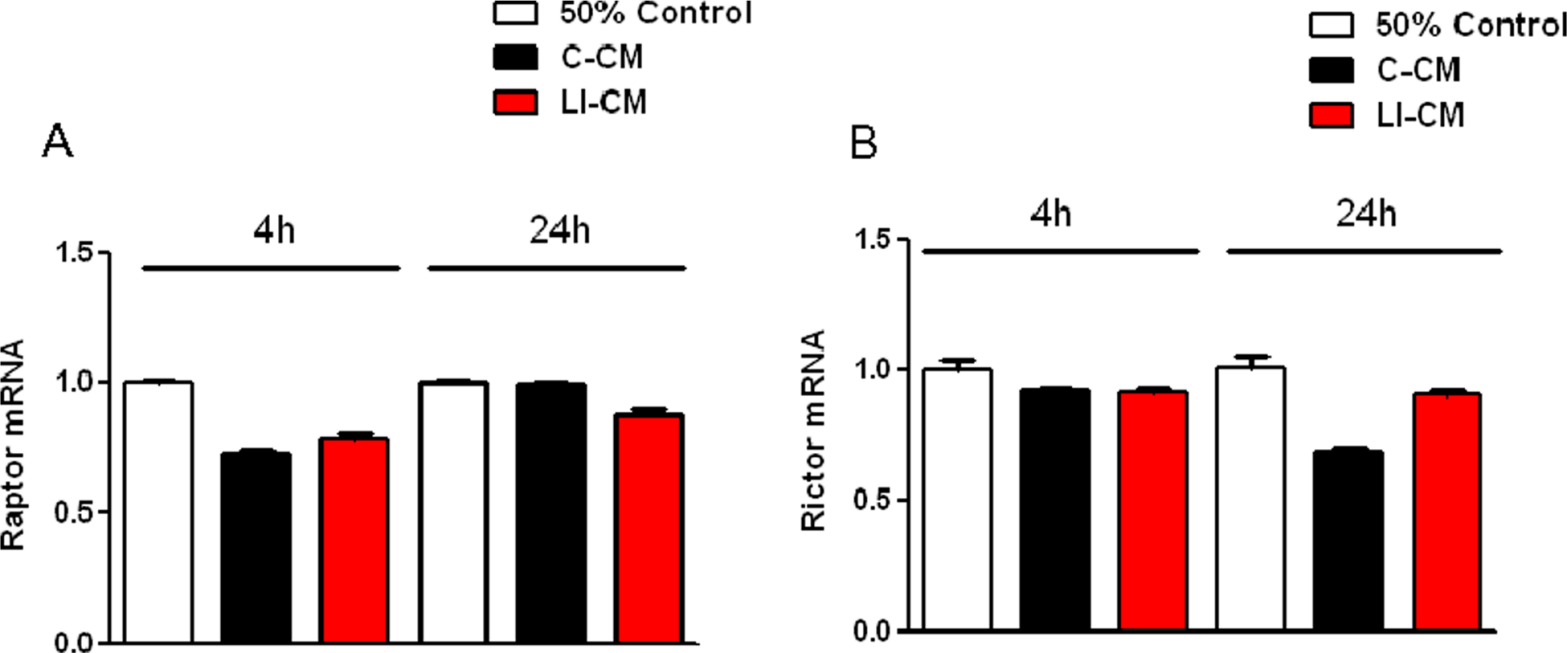
**Effects of the conditioned media on raptor and rictor expression.** Total cytosolic RNA was prepared from control, or microglial cells treated with control-conditioned medium (C-CM) or CM from glioma cells activated with lipopolysaccharide (LPS)/IFNγ (LI-CM) for different times, and used for real time (Q)-PCR analysis of raptor **(A)** and rictor **(B)** expression. Data are expressed as fold change versus control, taken as calibrator for comparative quantitation analysis of mRNA levels. Each sample was measured in triplicate and the experiment was repeated twice with similar results. Data are means ± standard error of the mean, and were analyzed by two-way analysis of variance followed by Bonferroni post hoc test.

**Figure 3:**
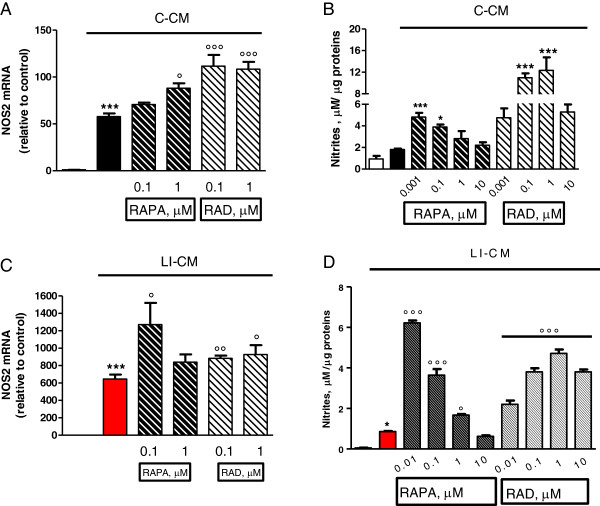
**Effects of mammalian target of rapamycin (mTOR) inhibitors on nitric oxide synthase (NOS)2 in microglia stimulated by both control-conditioned medium (C-CM) and CM from glioma cells activated with LPS/IFNγ (LI-CM).** Cells were treated with C-CM alone or in combination with mTOR inhibitors. **(A**-**B)** and with LI-CM alone or in combination with mTOR inhibitors **(C**-**D)**. Total cytosolic RNA was prepared from control, or microglial cells treated with the drugs for 4 h, and used for real time (Q)-PCR analysis of NOS2 expression. Data are expressed as fold change versus control, taken as calibrator for comparative quantitation analysis of mRNA levels. Each sample was measured in triplicate and the experiment was repeated three times with similar results. Data are means ± standard error of the mean (SEM), and were analyzed by one-way analysis of variance followed by Bonferroni post hoc test. ***P <0.001 versus control; °*P* <0.05 °°°*P* <0.001 versus C-CM and °*P* <0.05 °°*P* <0.01 versus LI-CM. **(B**-**D)** After 48-h incubation, the medium was used for nitrite assessment, whereas cells were lysed in 200 mM NaOH and protein content was evaluated by the Bradford method. Results are expressed as μM of nitrites/μg of proteins; data are means ± SEM (n = 6). *P <0.05, ***P <0.001 versus C-CM; *P <0.05 versus control and °*P* <0.001 °°°*P* <0.001 versus LI-CM.

**Figure 4:**
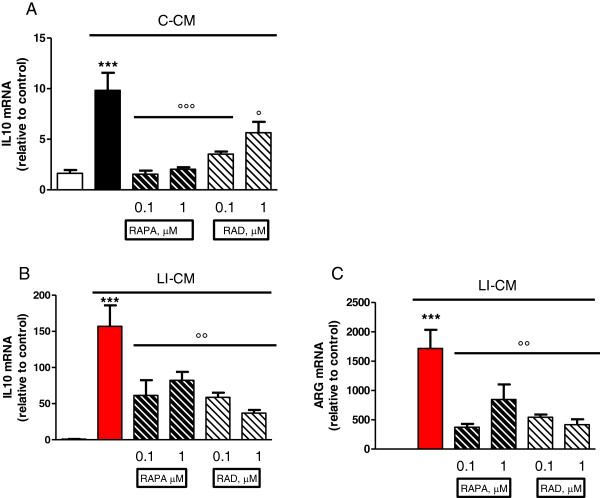
**Effects of mammalian target of rapamycin (mTOR) inhibitors on M2 markers in microglia stimulated by both control-conditioned medium (C-CM) and LI-CM.** Cells were treated with control-conditioned medium (C-CM) or CM from glioma cells activated with lipopolysaccharide (LPS)/IFNγ (LI-CM) and mTOR inhibitors. Total cytosolic RNA was prepared from control, or microglial cells treated with C-CM alone or with mTOR inhibitors for 4 h **(A)** and with LI-CM alone or with mTOR inhibitors for 4 h **(B)** or 24 h **(C)**, and used for real time (Q)-PCR analysis of IL-10 **(A**-**B)** and arginase (ARG)1 **(C)** expression. Data are expressed as fold change versus control, taken as calibrator for comparative quantitation analysis of mRNA levels. Each sample was measured in triplicate and the experiment was repeated three times with similar results. Data are means ± standard error of the mean, and were analyzed by one-way analysis of variance followed by Bonferroni post hoc test. ***P <0.001 versus control and °°°*P* <0.001, °*P* <0.05, versus C-CM; ***P <0.001 versus control and °°*P* <0.01 versus LI-CM.

We have previously shown that CM harvested from glioma cells under basal conditions (C-CM), as well as CM from glioma cells activated with LPS/IFNγ (LI-CM), increased the phosphorylation of mTOR at ser 2448, an index of mTORC activation, in microglial cells in 2-h experiments [21]. Here we confirmed this finding, and also observed that mTOR activation is rapid and time-persistent: after 30 minutes of incubation with both conditioned media we detected an increase in phospho-mTOR levels that persisted until 8 h (Figure 1). However, no significant upregulation was found in the mRNA levels of specific interactors of mTORC1 (raptor, Figure 2A) and mTORC2 (rictor, Figure 2B) in 4- and 24-h experiments.The administration of mTOR inhibitors (RAPA and RAD) to microglial cells exposed to C-CM significantly increased iNOS gene expression after 4 h (Figure 3A), followed by an increase in the levels of NO after 48 h (Figure 3B). Furthermore, RAPA and RAD were also able to increase iNOS mRNA levels after 4 h in microglial cells exposed to LI-CM (Figure 3C); such upregulation was followed by a significant augmentation of NO production after 48 h (Figure 3D).In addition, both drugs reduced the upregulation of IL-10 gene expression elicited by C-CM, with significant inhibition from 4 h onward (Figure 4A). In parallel, the drugs were able to reduce the upregulation of IL-10 (Figure 4B) and arginase (ARG) (Figure 4C) elicited by LI-CM. Taken together, these data show that mTOR blockade promotes M1 microglial polarization in both experimental paradigms (that is, conditions resembling the early stage of pathology and conditions mimicking the late stage of pathology).

**Figure 5:**
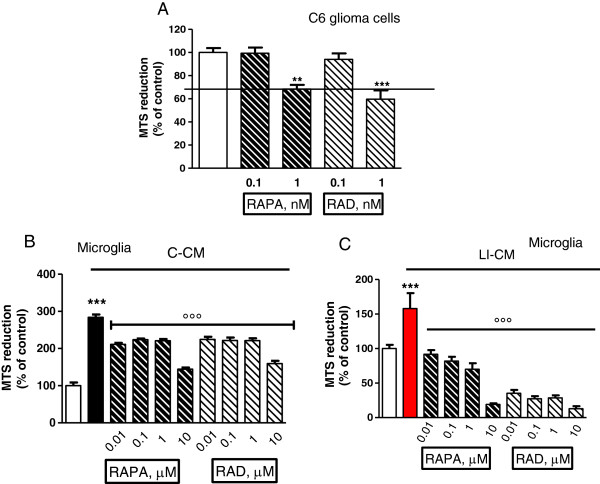
**Effects of mammalian target of rapamycin (mTOR) inhibitors on cell viability. (A)** C6 glioma cells were administrated with mTOR inhibitors. Microglia cells were treated for 48 h with control-conditioned medium (C-CM) **(B)** or CM from glioma cells activated with lipopolysaccharide (LPS)/IFNγ (LI-CM) **(C)** and mTOR inhibitors. Effects on cell viability were assessed by the MTS reduction assay. Data were analyzed by one-way analysis of variance followed by Bonferroni post hoc test. ***P <0.001, versus Control, °°°*P* <0.001 versus C-CM or LI-CM.

**Figure 6:**
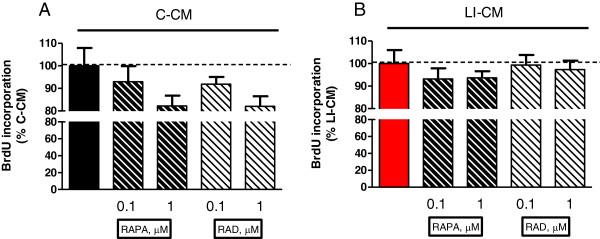
**Effects of mammalian target of rapamycin (mTOR) inhibitors on microglial proliferation.** Microglia cells were treated for 48 h with control-conditioned medium (C-CM) **(A)** or CM from glioma cells activated with lipopolysaccharide (LPS)/IFNγ (LI-CM) **(B)** and mTOR inhibitors. Effects on cell viability were assessed by the MTS reduction assay, while the effects on cell proliferation were assessed by 5-bromo-2-deoxyuridine (BrDU) incorporation. Data were analyzed by one-way analysis of variance followed by Bonferroni post hoc test.

A subsequent set of experiments was carried out to assess the effect of mTOR inhibitors on cell viability and proliferation. First, we tested the direct effects of RAPA and RAD on C6 glioma cells and confirmed that both drugs reduce glioma viability (Figure 5A), as reported in the literature. In particular, RAPA and RAD at 1 nM, reduced C6 glioma viability by about 30% after 3 days of treatment. Thereafter, we assessed the effect of mTOR inhibitors on microglia viability in cells exposed to glioma-conditioned media. In this experimental model, RAPA and RAD significantly counteracted the increase in cell viability induced by the exposure to C-CM or LI-CM, although significantly higher concentrations (approximately 1,000-fold higher) were required to achieve a 50% reduction with respect to those found effective on C6 glioma cells (Figure 5B and C) or else on microglial cells not exposed to glioma factors [13]. In untreated microglia, viability was significantly affected by mTOR inhibition even at a low dose (from 10 nM concentration onward) [13]. No significant differences were observed in microglia proliferation in the presence of mTOR inhibitors in the two different experimental paradigms (Figure 6).

## Discussion

In this study we evaluated the effects of mTOR inhibitors on microglial polarization in experimental models of glioma-microglia interaction. We have compared the effects of RAPA and its analog RAD on primary rat microglia activated by conditioned medium harvested from C6 glioma cultures in two different conditions. Drugs were administrated at high doses (0.01 to 10 μM concentrations), attempting to block the activity of both mTOR complexes. In fact, it was previously shown that RAPA given at higher concentrations and in chronic treatments also interferes with mTORC2 regulatory functions [26]. In particular, high intracellular levels of RAPA inhibit the binding and subsequent assembly of mTORC2-specific components Sin1 and rictor [24]. Rictor is a core component of mTORC2; we found that rictor is expressed in microglia cells, albeit its expression is not modulated by the exposure to either LI-CM or C-CM glioma-conditioned media (Figure 2B). Consistently with the literature, we found that microglial cells express the specific component of mTORC1, raptor, whose expression was also unaffected by the exposure to glioma factors (Figure 2A).

The crosstalk among mTORC1 and mTORC2 occurring in microglia cells ensures a correct balance between cell growth and division, regulating important intracellular metabolic processes and possibly different types of activation, as it is observed in other immune cells [29]. The activation of mTORC1 generally increases the cellular capacity of protein and lipid biosynthesis, and inhibits macroautophagy, thus promoting anabolic processes [1]. Hence it is not surprising that mTORC1 is deregulated in many tumor types, including malignant gliomas [11]. Several oncogenes (PI3K, AKT or Ras) and/or tumor suppressors (PTEN, LKB1, or TSC1/2), which are often mutated in cancer, contribute to control mTORC1 activation [3]. mTORC1 and mTORC2 are also important regulators of tumor invasiveness and cell migration [5,30]. mTORC2 activity is elevated in gliomas and promotes growth and cell motility via the overexpression of rictor [31], although it has been reported that rictor silencing by siRNAs in human glioma cell lines and primary GBM cells induces MMP-9 activity and enhances the invasive potential of glioma cells through the Raf-1-MEK-ERK signaling pathway [32].

The available evidence on the role of microglia in CNS cancers would suggest a dual role, a tumoricidal role and, on the contrary, a role favoring tumor growth, as described in detail in the Introduction section. A further viewpoint arises from the recent finding that subpopulations of cells within human gliomas, specifically GBM, are neoplastic macrophages/microglia [33]. Metastatic cancer cells retrieved in spontaneous mouse brain tumors appear to express multiple properties of macrophages [34], thus suggesting that the most aggressive cells in GBM may be neoplastic microglia/macrophages. In this regard, fusion of tumor cells with microglial cells has been proposed as a mechanism underlying invasion and metastasis in GBM, as it occurs in several human cancers [33]. In this scenario, data presented in this paper suggest that the inhibition of mTOR in microglial cells, apart from the more relevant antitumor effects mediated by the polarization of microglia towards an M1 phenotype (Figure 3), while on the same time polarization towards the M2 status is prevented (Figure 4), might also result in anti-proliferative activity directly on neoplastic microglia.

Microglial cells in the M1 status release proinflammatory cytokines, proteinases, complement proteins, prostaglandins, and reactive oxygen intermediates, including nitric oxide. These substances can exert toxic effects on glioma cells and hence contribute to their destruction. Nitric oxide, for example, has emerged as a powerful adjuvant for the hyper-sensitization of tumors to more traditional chemo- and radio-therapeutics. Furthermore, emerging evidence indicates that nitric oxide donors have the potential to exert intrinsic antitumor activity in the clinical management of cancer [35]. However, while the above discussion would suggest an overall favorable role for M1 polarization of microglia, it should be taken into account that the latter may also have detrimental effects, as it has been shown that chronic activation of microglia can cause damage to normal neurons through the release of the same substances that are potentially cytotoxic for glioma cells [36].

In conclusion, the main finding of this study is the observation that mTOR inhibition by RAPA and RAD prevents microglial polarization towards an M2 phenotype in *in vitro* models of both early- and late-stage glioma. The subsequent increase in the ratio of M1 microglial cells bearing cytotoxic and perhaps tumoricidal properties is a mechanism of potential interest in light of a possible clinical development of mTOR inhibitors in the treatment of human glioma.

## Abbreviations

ANOVA: analysis of variance; ARG: arginase; BMP: bone morphogenetic protein; BrdU: 5-bromo-2-deoxyuridine; C-CM: control-conditioned medium; CNS: central nervous system; Ct: threshold cycle; DMEM: Dulbecco's modified Eagle's medium; ELISA: enzyme-linked immunosorbent assay; FCS: fetal calf serum; GBM: glioblastoma; IFNγ: interferon-γ; IL: interleukin; iNOS: inducible nitric oxide synthase; LI-CM: inflammatory activated glioma cells; LPS: lipopolysaccharide; mTOR: mammalian target of rapamycin; PBS: phosphate buffered saline; RAPA: rapamycin; RAD: rapamycin analog RAD001; RTK: receptor tyrosine kinase; TSC: tuberous sclerosis complex; WHO: World Health Organization.

## Competing interests

The authors have no conflicts of interest to disclose.

## Authors’ contributions

Study concepts: LL, CDR, and PN; study design: LL, and EL; data acquisition: LL; data analysis and interpretation: LL, EL, and CDR; statistical analysis: LL; manuscript preparation: LL; manuscript editing: CDR; manuscript review: PN. All authors have read and approved the final version of the manuscript.

## References

[B1] Laplante M Sabatini DM mTOR signaling in growth control and disease Cell 2012 149 274 293 22500797 10.1016/j.cell.2012.03.017 PMC3331679

[B2] Foster KG Fingar DC Mammalian target of rapamycin (mTOR): conducting the cellular signaling symphony J Biol Chem 2010 285 14071 14077 20231296 10.1074/jbc.R109.094003 PMC2863215

[B3] Li J Kim SG Blenis J Rapamycin: One Drug, Many Effects Cell Metab 2014 19 373 379 24508508 10.1016/j.cmet.2014.01.001 PMC3972801

[B4] Menon S Manning BD Common corruption of the mTOR signaling network in human tumors Oncogene 2008 27 Suppl 2 43 51 10.1038/onc.2009.352 PMC3752670 19956179

[B5] Oh WJ Jacinto E mTOR complex 2 signaling and functions Cell Cycle 2011 10 2305 2316 21670596 10.4161/cc.10.14.16586 PMC3322468

[B6] Rolle CE Sengupta S Lesniak MS Mechanisms of immune evasion by gliomas Adv Exp Med Biol 2012 746 53 76 22639159 10.1007/978-1-4614-3146-6_5

[B7] Bradley D Rees J Updates in the management of high-grade glioma J Neurol 2014 261 651 654 23857040 10.1007/s00415-013-7032-x

[B8] Grzmil M Hemmings BA Overcoming resistance to rapalogs in gliomas by combinatory therapies Biochim Biophys Acta 2013 1834 1371 1380 23395884 10.1016/j.bbapap.2013.01.041

[B9] Krueger DA Care MM Holland K Agricola K Tudor C Mangeshkar P Wilson KA Byars A Sahmoud T Franz DN Everolimus for subependymal giant-cell astrocytomas in tuberous sclerosis N Engl J Med 2010 363 1801 1811 21047224 10.1056/NEJMoa1001671

[B10] Benjamin D Colombi M Moroni C Hall MN Rapamycin passes the torch: a new generation of mTOR inhibitors Nat Rev Drug Discov 2011 10 868 880 22037041 10.1038/nrd3531

[B11] Cloughesy TF Cavenee WK Mischel PS Glioblastoma: from molecular pathology to targeted treatment Annu Rev Pathol 2014 9 1 25 23937436 10.1146/annurev-pathol-011110-130324

[B12] Wander SA Hennessy BT Slingerland JM Next-generation mTOR inhibitors in clinical oncology: how pathway complexity informs therapeutic strategy J Clin Invest 2011 121 1231 1241 21490404 10.1172/JCI44145 PMC3069769

[B13] Dello Russo C Lisi L Tringali G Navarra P Involvement of mTOR kinase in cytokine-dependent microglial activation and cell proliferation Biochem Pharmacol 2009 78 1242 1251 19576187 10.1016/j.bcp.2009.06.097

[B14] Lisi L Navarra P Feinstein DL Dello Russo C The mTOR kinase inhibitor rapamycin decreases iNOS mRNA stability in astrocytes J Neuroinflammation 2011 8 1 21208419 10.1186/1742-2094-8-1 PMC3025854

[B15] Dello Russo C Lisi L Feinstein DL Navarra P mTOR kinase, a key player in the regulation of glial functions: relevance for the therapy of multiple sclerosis Glia 2013 61 301 311 23044764 10.1002/glia.22433

[B16] da Fonseca AC Badie B Microglia and macrophages in malignant gliomas: recent discoveries and implications for promising therapies Clin Dev Immunol 2013 2013 264124 23864876 10.1155/2013/264124 PMC3707269

[B17] Schwartz M Butovsky O Kipnis J Does inflammation in an autoimmune disease differ from inflammation in neurodegenerative diseases? Possible implications for therapy J Neuroimmune Pharmacol 2006 1 4 10 18040786 10.1007/s11481-005-9010-2

[B18] Mantovani A Sozzani S Locati M Allavena P Sica A Macrophage polarization: tumor-associated macrophages as a paradigm for polarized M2 mononuclear phagocytes Trends Immunol 2002 23 549 555 12401408 10.1016/s1471-4906(02)02302-5

[B19] Biswas SK Allavena P Mantovani A Tumor-associated macrophages: functional diversity, clinical significance, and open questions Semin Immunopathol 2013 35 585 600 23657835 10.1007/s00281-013-0367-7

[B20] Komohara Y Ohnishi K Kuratsu J Takeya M Possible involvement of the M2 anti-inflammatory macrophage phenotype in growth of human gliomas J Pathol 2008 216 15 24 18553315 10.1002/path.2370

[B21] Li W Graeber MB The molecular profile of microglia under the influence of glioma Neuro Oncol 2012 14 958 978 22573310 10.1093/neuonc/nos116 PMC3408253

[B22] Lisi L Stigliano E Lauriola L Navarra P Dello Russo C Proinflammatory-activated glioma cells induce a switch in microglial polarization and activation status, from a predominant M2b phenotype to a mixture of M1 and M2a/b polarized cells ASN Neuro 2014 6 171 183 24689533 10.1042/AN20130045 PMC4013688

[B23] Byles V Covarrubias AJ Ben-Sahra I Lamming DW Sabatini DM Manning BD Horng T The TSC-mTOR pathway regulates macrophage polarization Nat Commun 2013 4 2834 24280772 10.1038/ncomms3834 PMC3876736

[B24] Rocher C Singla DK SMAD-PI3K-Akt-mTOR pathway mediates BMP-7 polarization of monocytes into M2 macrophages PLoS One 2013 8 e84009 24376781 10.1371/journal.pone.0084009 PMC3869858

[B25] Mercalli A Calavita I Dugnani E Citro A Cantarelli E Nano R Melzi R Maffi P Secchi A Sordi V Piemonti L Rapamycin unbalances the polarization of human macrophages to M1 Immunology 2013 140 179 190 23710834 10.1111/imm.12126 PMC3784164

[B26] Sarbassov DD Ali SM Sengupta S Sheen JH Hsu PP Bagley AF Markhard AL Sabatini DM Prolonged rapamycin treatment inhibits mTORC2 assembly and Akt/PKB Mol Cell 2006 22 159 168 16603397 10.1016/j.molcel.2006.03.029

[B27] Dello Russo C Boullerne AI Gavrilyuk V Feinstein DL Inhibition of microglial inflammatory responses by norepinephrine: effects on nitric oxide and interleukin-1beta production J Neuroinflammation 2004 1 9 15285793 10.1186/1742-2094-1-9 PMC500870

[B28] Lisi L Tramutola A Navarra P Dello Russo C Antiretroviral agents increase NO production in gp120/IFNγ-stimulated cultures of rat microglia via an arginase-dependent mechanism J Neuroimmunol 2014 266 24 32 24268674 10.1016/j.jneuroim.2013.10.013

[B29] Powell JD Pollizzi KN Heikamp EB Horton MR Regulation of immune responses by mTOR Annu Rev Immunol 2012 30 39 68 22136167 10.1146/annurev-immunol-020711-075024 PMC3616892

[B30] Zhou H Huang S Role of mTOR signaling in tumor cell motility, invasion and metastasis Curr Protein Pept Sci 2011 12 30 42 21190521 10.2174/138920311795659407 PMC3410744

[B31] Masri J Bernath A Martin J Jo OD Vartanian R Funk A Gera J mTORC2 activity is elevated in gliomas and promotes growth and cell motility via overexpression of rictor Cancer Res 2007 67 11712 11720 18089801 10.1158/0008-5472.CAN-07-2223

[B32] Das G Shiras A Shanmuganandam K Shastry P Rictor regulates MMP-9 activity and invasion through Raf-1-MEK-ERK signaling pathway in glioma cells Mol Carcinog 2011 50 412 423 21557327 10.1002/mc.20723

[B33] Huysentruyt LC Akgoc Z Seyfried TN Hypothesis: are neoplastic macrophages/microglia present in glioblastoma multiforme? ASN Neuro 2011 3 e00064 21834792 10.1042/AN20110011 PMC3178415

[B34] Huysentruyt LC Mukherjee P Banerjee D Shelton LM Seyfried TN Metastatic cancer cells with macrophage properties: evidence from a new murine tumor model Int J Cancer 2008 123 73 84 18398829 10.1002/ijc.23492

[B35] Reynolds MM Witzeling SD Damodaran VB Medeiros TN Knodle RD Edwards MA Lookian PP Brown MA Applications for nitric oxide in halting proliferation of tumor cells Biochem Biophys Res Commun 2013 431 647 651 23337501 10.1016/j.bbrc.2013.01.041

[B36] Dheen ST Kaur C Ling EA Microglial activation and its implications in the brain diseases Curr Med Chem 2007 14 1189 1197 17504139 10.2174/092986707780597961

